# Group mating in Cretaceous water striders

**DOI:** 10.1098/rspb.2023.2546

**Published:** 2024-04-03

**Authors:** Yanzhe Fu, Chenyang Cai, Pingping Chen, Qiang Xuan, Tin Aung Myint, Diying Huang

**Affiliations:** ^1^ State Key Laboratory of Palaeobiology and Stratigraphy, Nanjing Institute of Geology and Palaeontology, and Centre for Excellence in Life and Palaeoenvironment, Chinese Academy of Sciences, Nanjing 210008, People's Republic of China; ^2^ Department of Biology II, Ludwig-Maximilians-Universität München, Biocenter, Großhaderner Str. 2, 82152 Planegg-Martinsried, Germany; ^3^ Section Entomology, Naturalis Biodiversity Centre, 2300 RA Leiden, The Netherlands; ^4^ Department of Geology, University of Mandalay, Mandalay, Myanmar

**Keywords:** mating behaviour, group mating fossil, sexual conflict, Myanmar amber, palaeoecology

## Abstract

Fossilized mating insects are irreplaceable material for comprehending the evolution of the mating behaviours and life-history traits in the deep-time record of insects as well as the potential sexual conflict. However, cases of mating pairs are particularly rare in fossil insects, especially aquatic or semi-aquatic species. Here, we report the first fossil record of a group of water striders in copulation (including three pairs and a single adult male) based on fossils from the mid-Cretaceous of northern Myanmar. The new taxon, *Burmogerris* gen. nov., likely represents one of the oldest cases of insects related to the marine environment, such as billabongs formed by the tides. It exhibits conspicuous dimorphism associated with sexual conflict: the male is equipped with a specialized protibial comb as a grasping apparatus, likely representing an adaptation to overcome female resistance during struggles. The paired *Burmogerris* show smaller males riding on the backs of the females, seemingly recording a scene of copulatory struggles between the sexes. Our discovery reveals a mating system dominated by males and sheds light on the potential sexual conflicts of *Burmogerris* in the Cretaceous. It indicates the mating behaviour remained stable over long-term geological time in these water-walking insects.

## 1. Introduction

The semi-aquatic hemipteran infraorder Gerromorpha Popov [1] comprise water striders and their allies, which are conspicuously adapted to motion, feeding and mating on the water surface, occupying a vast array of niches including freshwaters, lagoons, sea near the coasts and even the pelagic environments [2,3]. The specialized morphology and diverse behaviour of gerromorphans have become hotspots in evolutionary biology, ecology and even interdisciplinary studies, and provided a theoretical basis for developing biomimetic technology on the surface of water [4–6]. The largest gerromorphan clade is Gerridae Leach [7] (water striders) + Veliidae Amyot & Audinet-Serville [8] (broad-shouldered water striders) [9,10]. In comparison to the diversity of extant species, the Gerridae has a restricted fossil record [9,11]. Except for the only example of Mesozoic Gerridae from Late Albian French amber [12], other fossil gerrids are documented in the Cenozoic deposits of China, Europe and North America (Eocene–Miocene) [11,13–17]. Similarly, the fossil records of the Veliidae are extremely scarce, exclusively found in amber bioinclusions [15,16,18–20].

Sexual conflict occurs widely in various animal species [21–24] and is considered a significant driver of male–female coevolution in insects, with profound implications for the evolution of reproductive isolation and speciation [22,23,25]. Gerromorphans constitute a classical model for the study of sexual selection and sexual conflict [26–30]. The most common mating system in extant water striders and relatives is characterized by strong sexual conflicts, manifested as copulatory struggles between the sexes, together with a series male behaviours such as harassment, coercive violation and intimidation, while females resist costly mating attempts [21,22,26,27,29,31–34]. The large-sized *Gerris gracilicornis* (Horváth, 1879) [35] male even employs an intimidating signalling strategy by directly attracting underwater predators to punish females that reject copulation attempts [29,36].

Currently, understanding the origin and evolution of sexual conflict in insects is hampered by the scarcity of fossilized mating cases [37]. To date, only two cases of mating behaviour in fossil gerromorphan bugs have been reported, both found in Dominican amber [37]. These cases involved a pair of broad-shouldered water striders preserved in a mating posture [18], and a couple of water striders that were interpreted as postcopulatory guarding [13]. Here, we report the discovery of paired fossil water striders in groups and describe a new taxon, *Burmogerris rarus* gen. et sp. nov., from mid-Cretaceous amber of northern Myanmar, allowing us to explore mating strategies and potential sexual conflict among water striders *ca* 100 Ma.

## 2. Material and methods

The studied amber sample measures approximately 40 mm in length, 21 mm in width and 8.5 mm in thickness, with a weight of about 13 g. The amber piece in this study originated from amber mines near Noije Bum Hill, Hukawng Valley, Myitkyina District of Kachin State, northern Myanmar. Available data suggest that the age of the Burmese amber was generally assigned to be around the Albian–Cenomanian boundary [38–40]. The studied amber specimen is deposited in Nanjing Institute of Geology and Palaeontology, Chinese Academy of Sciences, Nanjing, China.

The amber piece was polished with different grades of sandpaper of gradually finer grits, and finally with rare earth polishing powder. Bright-field images were taken with a Zeiss Discovery V16 stereo microscope. The Zeiss Axio Imager 2 microscope was equipped with a mercury lamp and specific filters for DAPI, eGFP and rhodamine. Photomicrographs with a green background were taken under the eGFP mode (Zeiss Filter Set 10; excitation/emission: 450–490/515–565 nm). Fluorescence images were converted to greyscale to enhance the visibility of structures. Confocal images were obtained with a Zeiss LSM710 confocal laser scanning microscope (CLSM), using the 488 nm Argon laser excitation line [41]. Focus stacking software (Helicon Focus 7.0.2) was used to increase the depth of field. Morphological measurements were conducted using ImageJ software.

## 3. Results

### (a) Systematic palaeontology

Infraorder Gerromorpha Popov, 1971

Superfamily Gerroidea Leach, 1815

Family incertae sedis

*Burmogerris* Fu, Cai, Chen & Huang, gen. nov.

urn:lsid:zoobank.org:act:E955D12B-DA7A-4897-84DF-FB335FEBF215

#### (i) Type species

*Burmogerris rarus* Fu, Cai, Chen & Huang, sp. nov.; by present designation.

#### (ii) Etymology

The generic name is a combination of the prefix ‘Burma-’ referring to Myanmar, and *Gerris*, the type genus of the family Gerridae. Gender masculine.

#### (iii) Diagnosis

The genus is characterized by a combination of the following characters: macropterous; head without median impressed line on the dorsal surface (a typical median line on the dorsal surface of head in veliids); antennal segment II elongate, slightly shorter than segment I and more than twice as long as two apical segments, segment IV widened, less spindle-shaped. All tarsi three-segmented (two-segmented in all gerrids), first tarsomere extremely short, subcylindrical; forelegs prolonged, almost as long as body length; male protibia bearing numerous discontinuous clusters of pegs (absent in male gerrids); protarsus more than one-half length of the protibia, with second tarsomere about 1.20 times as long as the apical tarsomere; mesotibia bearing a row of long and slender trichobothria-like hairs; mesofemur as long as or slightly longer than the mesotibia; mesotibia almost as long as the mesotarsus; metatarsus with the second tarsomere much longer than the apical tarsomere; claws inserted on the apex of apical tarsomere; arolia absent.

**Figure 1.**
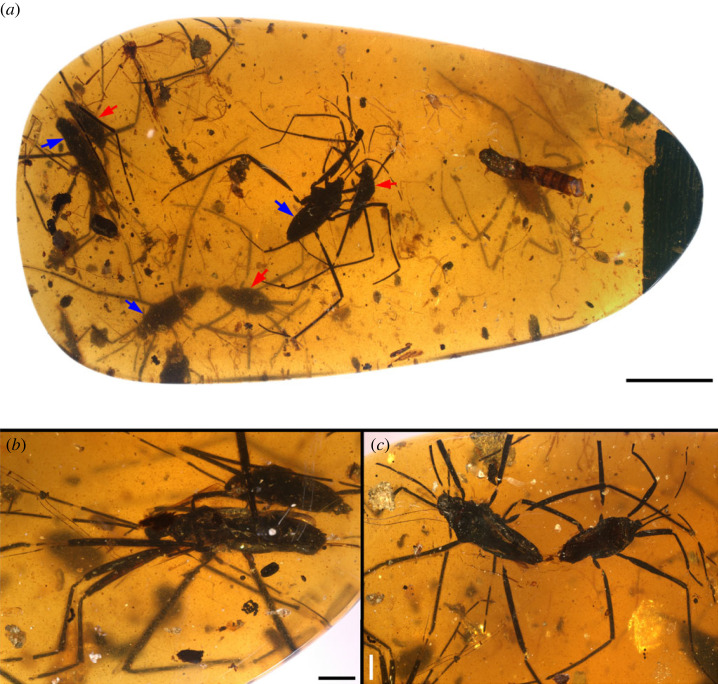
The paired water striders *Burmogerris rarus* gen. et sp. nov., from mid-Cretaceous Burmese amber, under bright-field microscopy. (*a*) An overall view of the amber piece; red and blue arrows indicate adult males and females, respectively; the middle pair consists of the holotype (NIGP201886, male) and the allotype (NIGP201887, female). (*b*) Paratypes, NIGP201888 and NIGP201889. (*c*) Paratypes, NIGP201890 and NIGP201891. Scale bars: 5 mm in (*a*), 1 mm in (*b,c*).

**Figure 2.**
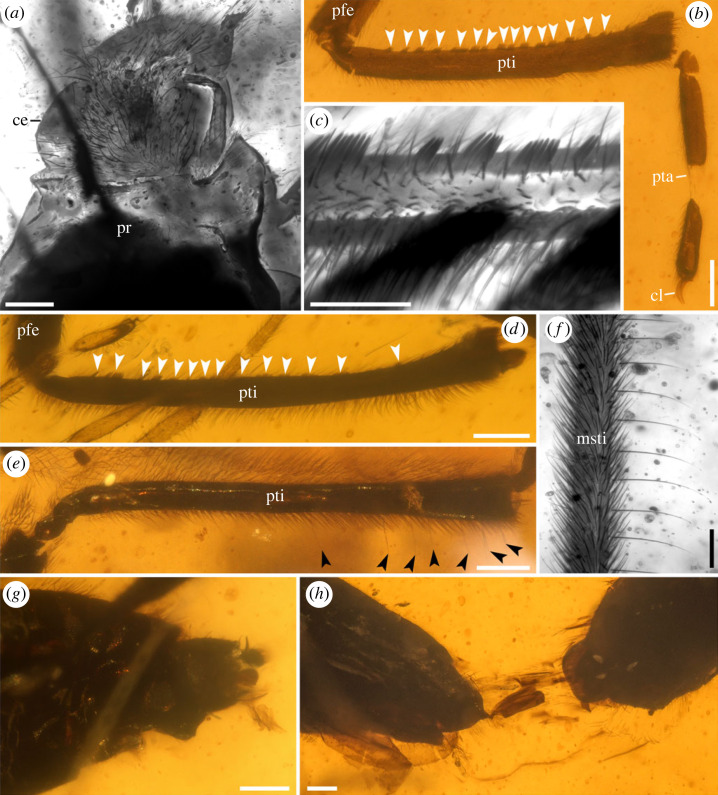
Photomicrographs of *Burmogerris rarus* gen. et sp. nov. (*a*) Head and pronotum (NIGP201891). (*b*) Protibia and protarsus (NIGP201892), write arrows indicate each cluster of pegs. (*c*) Protibial grasping comb (NIGP201886). (*d*) Protibia of male (NIGP201886), write arrows indicate each cluster of pegs. (*e*) Protibia of female (NIGP201889), black arrows indicate the trichobothria-like hairs. (*f*) Trichobothria-like hairs on mesotibia (NIGP201889). (*g*) Male genitalia (NIGP201888). (*h*) Male genitalia (NIGP201890) and female genitalia (NIGP201891). (*a,c,f*) Under confocal microscopy; (*b,d,e,g,h*) under bright-field microscopy. ce, compound eye; pr, pronotum; pfe, profemur; pti, protibia; pta, protarsus; msti, mesotibia; cl, claw. Scale bars: 0.2 mm in (*a,b,d,e,g,h*), 0.1 mm in (*c,f*).

*Burmogerris rarus* Fu, Cai, Chen & Huang, sp. nov.

urn:lsid:zoobank.org:act:D0265B95-D0E7-434A-9165-390AD3A87214

(figures 1 and 2; electronic supplementary material, figures S1–S5)

#### (iv) Etymology

The specific epithet ‘rarus’ (Latin, adjective, meaning rare) refers to some odd morphological characters and its rare existence.

#### (v) Type material

**Figure 3.**
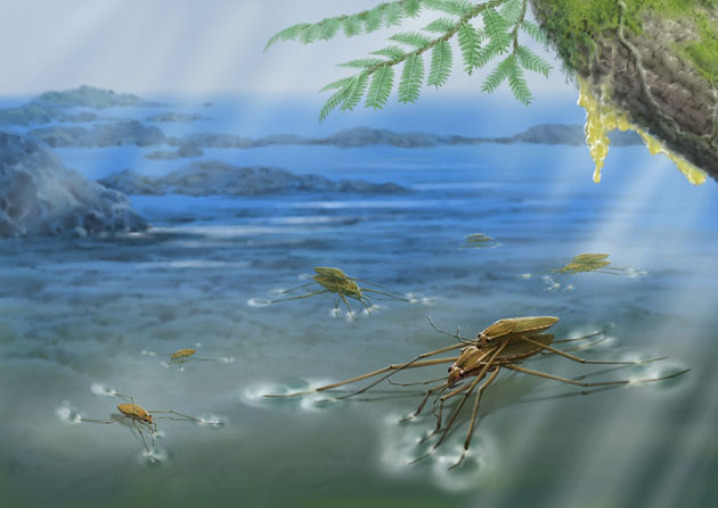
Ecological reconstruction of *Burmogerris rarus* gen. et sp. nov. in the Burmese amber forests during the mid-Cretaceous.

Holotype: NIGP201886 (adult male); allotype: NIGP201887 (adult female); paratypes: NIGP201888–NIGP201892 (adults), and NIGP203312–NIGP203315 (nymphs); mid-Cretaceous, from an amber mine near Noije Bum Village, Hukawng Valley, Tanai Township, Myitkyina District, Kachin State, northern Myanmar (figure 3).

#### (vi) Diagnosis

As for the genus.

#### (vii) Description

For a full description of *Burmogerris rarus* gen. et sp. nov., refer to the electronic supplementary material.

### (b) Amber inclusions

In the studied specimen, seven adults (four males and three females) of *B*. *rarus*, containing three paired individuals and a single adult male, are enclosed in a relatively opaque, orange amber piece (figure 1*a*). The total length (excluding antennae and legs) of females is *ca* 4.81–4.95 mm. The total length of the single male (NIGP201892) is *ca* 4.59 mm, whereas the paired males are relatively short, ranging from 3.49 to 3.82 mm. Considering their almost identical identifiable traits of antenna and legs, we tentatively assign these specimens to the same species. Female/male size ratios of these three pairs are between 1.26 and 1.42. Two pairs were apparently captured in mating situations, showing smaller males riding on the backs of the females, but without direct genital contact between the males and females (figure 1*a,b*). In the paired individuals of NIGP201886 (male) and NIGP201887 (female), the male body is positioned in front of the female, apparently above and not parallel to the female axis (figure 1*a*). In the paired individuals of NIGP201888 (male) and NIGP201889 (female), the male is positioned behind the female, orienting itself slightly above and subparallel to the female body axis, with the male's left protibia tightly attached to the female's thorax (figure 1*b*). In the remaining pair, NIGP201890 (male) and NIGP201891 (female) are very close to each other (figure 1*c*). Additionally, four recognizable nymphs and fragments of water striders (electronic supplementary material, figure S5), along with a culicid, a beetle larva, four mites, several plant trichomes, etc. (electronic supplementary material, figure S6), are preserved as synbioinclusions. All nymphs seem to be similar instar due to their similar sizes (*ca* 0.80–1.14 mm) and morphological traits. We consider it likely that they are the same species as the adults preserved nearby.

## 4. Discussion

### (a) Systematic position of *Burmogerris*

Morphologically, *Burmogerris* resembles Gerridae (macropterous forms) in the following characters: a less elongated body with long and slender legs, a head somewhat extending beyond the anterior margin of the eyes, the absence of an impressed median dorsal line and ocelli, elongate antennae with the first segment being the longest, and a labium that surpassed the prosternum with segment III being the longest [2,42]. However, *Burmogerris* cannot be classified in any known subfamilies of Gerridae as the leg traits confusingly show some similarities with ‘Veliidae’, but note that the family is probably paraphyletic [9,10]. Significantly, the tarsi appear to be three-segmented in *Burmogerris*, with the basal segment being very short (figure 2*b*; electronic supplementary material, figures S2*b,f* and S4*b,d*). The three-segmented tarsus is regarded as an ancestral state of ‘Veliidae’ + Gerridae, as found in Veliinae Amyot & Audinet-Serville [8], Ocelloveliinae Drake & Chapman [43], and most Rhagoveliinae China & Usinger [44], all of which are currently placed in ‘Veliidae' [2,42]. The presence of a large campaniform sensillum on the basal tarsal segment in Gerridae suggests that the basal tarsus is composed of the fused reduced basal and second segments [2]. By contrast, two-segmented tarsi are found in Gerridae and the remaining subfamilies of ‘Veliidae’ [2,42]. The subfamilies Microveliinae China & Usinger [44] and Haloveliinae Esaki [45] (currently placed in ‘Veliidae’) are considered to form a monophyletic clade, and the sister relationship between this clade and Gerridae has been supported by morphological synapomorphies [46] and a recent phylogenomic study [10]. A distinctive morphological synapomorphy of the clade Microveliinae + Haloveliinae is the fusion of tarsomeres I and II in all legs, with a tarsal formula of 1 : 2 : 2 for Microveliinae and 2 : 2 : 2 for Haloveliinae [10]. Lastly, *Burmogerris* distinctly differs from all known subfamilies of ‘Veliidae’ in the absence of a typical median line on the dorsal surface of the head and a pair of deep pits near the posterior corner of eyes (figure 2*a*). The median line, however, is indistinct in some Microveliinae and Haloveliinae, whereas in Rhagoveliinae, the presence of longitudinal rows of punctures along the midline [2].

Gerridae and some subfamilies currently assigned to ‘Veliidae’ lack convincing diagnostic characters [47]. The classification of ‘Veliidae’ has long been confused with Gerridae, and the relationships between Gerridae and the subfamilies of ‘Veliidae’ remain debatable [9,10]. Recent phylogenetic reconstruction based on transcriptomic data indicated that ‘Veliidae’ constitutes successive sister groups to Gerridae [10]. Given its distinctive combination of characteristics, we tentatively regard *Burmogerris* as family incertae sedis within Gerroidea.

### (b) The sexual conflict in the evolution of water striders

Sexual conflict between two sexes occurs when there are divergent reproductive interests, particularly concerning the mode, frequency and duration of copulation [22,23]. Sexual conflict, specifically sexual coercion, may drive the striking morphological modification and dimorphism [21,34,48,49]. For example, it is believed that copulatory struggles have driven the evolution of male grasping structures to overcome female resistance in water striders and relatives [27,33,34,50,51]. The new fossils exhibit conspicuous sexual dimorphism in the protibia: (1) the protibia is slightly curved in males (figure 2*b,d*; electronic supplementary material, figure S4*b,c*), whereas in females, the protibia is straight (figure 2*e*; electronic supplementary material, figure S2*a*); (2) the protibia of males is armed with 15–17 discontinuous clusters of pegs along the innermost edge, consisting of 2–12 in each cluster (figure 2*b–d*; electronic supplementary material, figures S2*c,d* and S4*b,c*), forming a comb-like structure that is absent in females (figure 2*e*; electronic supplementary material, figure S2*a*). Various specialized grasping traits, including modified pregenital abdominal segments and external genital structures [27,50,52–54], equipped and thickened profemora [2,27,32], and highly specialized antennae and hind legs that form clamps [2,33], have evolved in male gerrid species primarily for grasping and fighting during mating. In the related ‘Veliidae’, males commonly possess protibial combs as a clasping device [2,34,51]. The primary difference in protibial combs between the new fossil and most ‘Veliidae’ is that the pegs are continuously distributed in the latter. We propose that the specialized protibial combs in male *B*. *rarus* represents an adaptation associated with the control of females during mating. This potentially enhances the males' capability to firmly grasp reluctant females during copulatory struggles, suggesting that sexual conflict in water striders could be traced back to at least the mid-Cretaceous, with related evolutionary arms races between the sexes possibly occurring. Additionally, the body shape also exhibits sexual dimorphism in *B*. *rarus*. The somewhat flattened ventral surface of the male abdomen (figure 1*a,b*) likely represents an adaptation for a close fit to the female body during mating [33]. In modern groups of Gerridae and ‘Veliidae’, females have evolved a series of counter adaptations, including both morphological modifications and behavioural strategies, to enhance their control over mating [27,33,34]. It appears that the female *B*. *rarus* lacks morphologically modifications that effectively prevent forced copulation. Nevertheless, the pair of NIGP201890 and NIGP201891 exhibits a tail-to-tail posture (figure 1*c*), and the fragments of genital structure between the ends of their abdomens can be clearly observed (figure 2*h*), possibly indicating a scenario where the male is dislodged after the female's fierce resistance during the copulatory struggle. Similar precopulatory struggle scenes were observed in *Rhagovelia antilleana* Polhemus [55], where females vigorously shake their bodies and perform repeated backward somersaults to reject the male (electronic supplementary material, video S2 [34]). Therefore, females may have developed effective behaviours in their resistance arsenal, such as performing backward somersaults to forcefully dislodge males. Another possibility is that the pair's posture is simply a result of stress-related effects, caused by resin flow, perhaps even occurring during mating.

Notably, sexual conflict is supposed to have a significant effect on the evolution of mating behaviour in water striders [33]. The most common mating system (Type I matings) generally characterized by intense sexual conflicts between the sexes, including pre- and postcopulatory struggles [32,33]. Certainly, some gerrid species exhibit a seemingly developed mating system (Type II matings), characterized by the absence or reduced intensity of precopulatory struggles [32,33]. In this system, males typically exhibit territorial behaviour by producing ripple signals to court females and defending oviposition sites, such as floating plants [56,57]. Moreover, several gerrid species display varying male mating strategies that change as the breeding season progresses [58]. A pair of fossil water striders, *Electrobates spinipes* Andersen & Poinar [13], has been reported from the Miocene Dominican amber, interpreted as postcopulatory guarding, as evidence by the male positioned behind and grasping the female's metafemora with his fore legs [13]. Contact guarding is frequently observed in Type I matings in extant water striders [33]. In *E*. *spinipes*, the protibia appears widened in the middle, especially in males, and is equipped with a row of short spines in both sexes [13], with seemingly absent sex combs. In the presented amber, three paired individuals are closely associated (figure 1), likely capturing a scene of copulatory struggle, where males grip the setae on the females' thorax using their modified protibiae. Among them, two pairs and a single male appear to have been preserved on the same layer at the upper surface of the amber (electronic supplementary material, figure S7), suggesting they were probably trapped simultaneously, documenting a microevent. The amber is composed of distinct layers, each potentially representing resin flows occurring at various times [59]. The additional pair, located in the lowest layer, likely documented a separate microevent, potentially occurring minutes, hours, or even days apart [59]. However, all syninclusions present in consecutive layers might be spatially very close to each other. Therefore, we propose a potential interpretation that the small-sized male *B*. *rarus* might exhibit gregarious behaviour and is unlikely to be territorial, while maintaining a high population density in the Burmese amber forest. Males of *B*. *rarus* are more likely to actively search for females rather than adopt a sit-and-wait strategy. This likely represents a behavioural trait associated with typical Type I matings from this perspective, which has remained stable over long-term geological time in water striders. Nevertheless, it remains unclear whether these paired fossils represent a stage of precopulatory struggle or postcopulatory contact guarding. Guarding behaviour could result in an extended association between males and females, thereby increasing the probability of being trapped by resin during the mating process. Furthermore, it is conceivable that males would prolong mating to avoid sperm competition, especially in a habitat with high population density. The specific mating strategy of male *B*. *rarus* remains elusive and may even involve a more complex behavioural flexibility (e.g. a mixed strategies between Type I and II matings or potentially a distinct approach) during the Cretaceous.

### (c) Palaeoecology

The mid-Cretaceous Burmese amber harbours the most diverse Mesozoic palaeobiota [60,61]. Recently, a few organisms associated with a marine lifestyle, such as pholadid bivalves, gastropods and ammonite, and fragments of crinoid column ossicles, corals and oysters have been found attached to the outer surface of amber pieces, suggesting that the Burmese amber forest was situated close to a shifting coastal environment [40,62]. Furthermore, the discovery of aquatic organisms in Burmese amber, such as true crabs [63], shrimp [64], seed shrimps [65], and various aquatic and semi-aquatic insects [20,66,67], suggests the presence of abundant water bodies in the Burmese amber forest.

*Burmogerris rarus* is unlikely to be a pelagic or strictly marine species due to the absence of relevant morphological adaptations, such as the loss of wings, the short and broad body shape, and the shortened fore legs [2]. Its appearance distinctly differs from known modern species of sea skaters, which are unique in their adaptation to living permanently on open oceans [2,68]. In *B*. *rarus*, the midlegs are subequal in length to or slightly longer than the hind legs, being significantly longer than the body, which contributes to improve its locomotion on the water surface [69]. Nevertheless, gerrids that occupy open freshwater surface niches typically have midlegs that are apparently longer than their hind legs and a higher ratio of midleg length/body length. The legs of *B*. *rarus* are covered by two types of dense coat of hairs, especially on the tibiae, possibly to resist wetting. The claws of *B*. *rarus* are inserted at the apex of the apical tarsi, which is commonly interpreted as either an ancestral status or secondary adaption to a semi-aquatic habitat [2]. Thus, *B*. *rarus* could inhabit marginal aquatic environments or microhabitats with less stability, which, to some extent, explains why the copulatory specimens were trapped by resin. Based on our understanding of the palaeoecology of the Burmese amber biota, we suggest that *B*. *rarus*, being a small-sized species, most likely inhabited small water bodies with slow-flowing water near the seashore in the Burmese amber forest (figure 3), such as tide-formed pools where water salinity fluctuates. When small water bodies dry up, winged *B*. *rarus* could regularly fly to colonize new habitats. This indicates that the species may have evolved the ability to adapt to short-term salinity fluctuations.

In the present case, three paired water striders maintain a copulatory struggling posture and a culicid undergoing stress-induced oviposition (electronic supplementary material, figure S6*a*) in the same amber piece suggest that these organisms were trapped *in situ* by resin. Regarding the incomplete beetle larva (electronic supplementary material, figure S6*g*), it could be attributed to Ptilodactylidae as a synbioinclusion. Extant ptilodactylid larvae are commonly associated with riparian and aquatic habitats. One possibility in the presented assemblage of amber piece is that plant trichomes and lower-lying mites were initially trapped along with the flows of resin. Subsequently, the resin dropped into a pond under the resin-producing tree and barely solidifies [70], ultimately capturing the swarms of paired water striders, a culicid and a nearby beetle larva.

## Nomenclatural acts

This published work and the nomenclatural acts have been registered in ZooBank. The LSID for this publication is urn:lsid:zoobank.org:pub:1537D9B8-5D11-4C83-97CA-F5E0186BCC53.

## Data Availability

All data generated during this study are included in the supplementary materials [71].
